# Rebalancing the Skin: The Microbiome, Acne Pathogenesis, and the Future of Natural and Synthetic Therapies

**DOI:** 10.3390/molecules30244684

**Published:** 2025-12-07

**Authors:** Maria Beatriz Oliveira, Ana Colette Maurício, Ana Novo Barros, Cláudia Botelho

**Affiliations:** 1 Centre for Animal Science Studies (CECA), Institute of Sciences, Technologies and Agroenvironment (ICETA), University of Porto, 5050-313 Porto, Portugal; up202402415@up.pt (M.B.O.); acmauricio@icbas.up.pt (A.C.M.); 2 Associated Laboratory for Animal and Veterinary Science (AL4AnimalS), 1300-477 Lisbon, Portugal; 3 Department of Veterinary Clinic, School of Medicine and Biomedical Sciences (ICBAS), University of Porto, 5050-313 Porto, Portugal; 4 Centre for the Research and Technology of Agro-Environmental and Biological Sciences (CITAB), Inov4Agro, University of Trás-os-Montes and Alto Douro (UTAD), 5000-801 Vila Real, Portugal; abarros@utad.pt

**Keywords:** skin microbiome, acne vulgaris, *Cutibacterium acnes*, natural bioactives, retinoids, antimicrobial resistance, EGCG, resveratrol, one health

## Abstract

The skin serves as a primary interface between the human body and the external environment, functioning both as a protective barrier and as a habitat for a complex and diverse microbiome. These microbial communities contribute to immune regulation, barrier integrity, and defence against pathogens. Disruptions in this equilibrium can precipitate dermatological disorders such as acne vulgaris, which affects millions of adolescents and adults worldwide. This chronic inflammatory disorder of the pilosebaceous unit is driven by microbial dysbiosis, hyperkeratinisation, sebum overproduction, and inflammation. This review synthesizes data from over 100 sources to examine the interplay between the skin microbiome and acne pathogenesis, and to compare synthetic treatments, including retinoids, antibiotics, and hormonal therapies, with natural approaches such as polyphenols, minerals, and resveratrol. The analysis highlights the therapeutic convergence of traditional pharmacology and bioactive natural compounds, proposing microbiome-conscious and sustainable strategies for future acne management.

## 1. Introduction

The human skin is a multifunctional organ that operates as both a physical barrier and a dynamic biological ecosystem where host cells and microorganisms coexist in equilibrium. The skin microbiome refers to all microorganisms present on the skin and their collective genetic material, while the microbiota encompasses only the microorganisms themselves. This distinction matters, as microbial genes contribute metabolic and immunological capacities that complement the host genome, conferring adaptive advantages the human genome alone has not evolved to provide [1,2,3,4,5,6,7,8,9].

Healthy skin hosts a rich and diverse community comprising bacteria, fungi, viruses, and microscopic arthropods. These microbial communities form a structured ecosystem, with each organism occupying a niche defined by topography, humidity, temperature, sebum content, and pH. The skin stands out among epithelial surfaces for its complex ecological interactions with the external environment. Gram-positive species dominate, particularly *Cutibacterium*, *Staphylococcus*, and *Corynebacterium*, because their thick peptidoglycan walls provide high structural stability, enabling survival under desiccation, osmotic stress, and UV exposure [1,2,3,4,5,6,7,8,10].

Cutaneous microbes also play essential defensive and metabolic roles. They produce inhibitory substances—such as bacteriocins, enzymes, and low-molecular-weight antimicrobials—that prevent colonisation by pathogenic species. This ecological competition, both intra- and interspecific, contributes to maintaining a stable and resilient microbiome. The overall stability of the skin’s microbial community, even under environmental fluctuation, suggests long-term coevolution between host and microflora [8,9,11,12,13,14,15,16,17].

In healthy adults, pilosebaceous follicles and sebum-rich areas harbour abundant populations of *Cutibacterium acnes* (formerly *Propionibacterium acnes*) and related propionibacteria, which occupy ecological niches that might otherwise be colonized by pathogens. Under physiological conditions, these bacteria are either benign or beneficial; however, they may acquire pathogenic potential in response to trauma, immune dysregulation, or barrier disruption [5,16,17,18,19,20,21,22].

Homeostasis within the cutaneous ecosystem depends on a delicate balance between microbiota and host. Disruptions—whether endogenous (e.g., genetic variation) or exogenous (e.g., excessive washing, antibiotic exposure, or altered hygiene practices)—can precipitate dysbiosis and increase susceptibility to inflammatory or infectious dermatoses Individual differences in skin biochemistry, local physiology, and product use make it difficult to establish universal links, site physiology, and product use, establishing universal correlations between specific organisms and skin function is challenging [8,18,20,21,22,23,24,25].

Host–microbe relationships on the skin can be categorised as mutualistic, commensal, or parasitic. In mutualism, both partners benefit (for example, *Staphylococcus epidermidis* can stimulate host antimicrobial peptide production); in commensalism, one benefits without harming the other; and in parasitism, one benefits at the host’s expense. Microorganisms obtain nutrients and a stable environment, the host benefits from microbial metabolic flexibility and rapid evolutionary adaptability. These dynamics are central to the skin’s immune equilibrium [8,21,22,26,27,28].

Physiologically, the skin is relatively cool, acidic (average pH ≈ 5.0), and relatively dry, but it includes multiple microhabitats that vary in temperature, humidity, lipid composition, and antimicrobial milieu. Structural appendages such as hair follicles, sebaceous, eccrine, and apocrine glands generate distinct environments that shape microbial diversity. In healthy skin, nutrients such as amino acids, vitamins, lactate, and lipids derive from sweat and sebum. Elements of the innate immune system—including β-defensins (HBD-1, -2, -3) and dermcidin—help control microbial growth and modulate microbial population density. Dermcidin, secreted in sweat, functions optimally under native saline and acidic conditions and inhibits both Gram-positive and Gram-negative bacteria [5,29,30,31,32,33,34].

**Figure 1:**
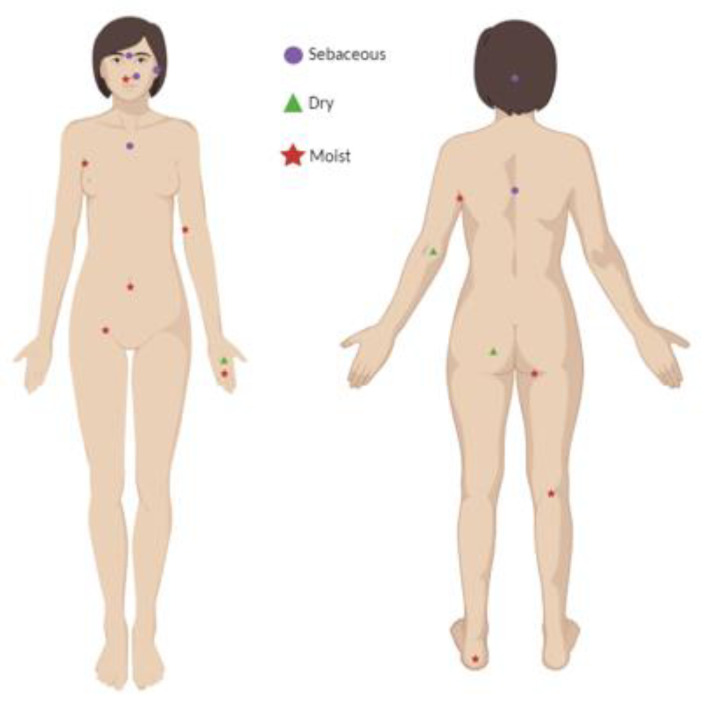
Identification of representative human skin regions with distinct physiological profiles (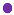 sebaceous, 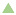 dry, and 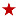 moist body sites) Created with BioRender.com.

Ecologically, the skin can be divided into three principal microenvironments—sebaceous, moist, and dry—each with characteristic taxa. Sebaceous regions (e.g., glabella, alar crease, external auditory canal, occiput, upper chest, and back) favour lipid-adapted speciessuch as *Cutibacterium* and *Malassezia*; moist regions (e.g., nares, axilla, antecubital fossa, interdigital spaces, inguinal crease, umbilicus, popliteal fossa, plantar heel) support *Staphylococcus* and *Corynebacterium*; dry sites (e.g., volar forearm, hypothenar palm, buttocks) exhibit greater diversity, including β-Proteobacteria and Flavobacteriales [1,23,34,35,36,37] (Figure 1).

Local physiochemistry further shapes colonisation. Fatty acids in sweat help maintain the acid mantle (pH ≈ 5), which inhibits *Staphylococcus aureus* and *Streptococcus pyogenes*, while occlusion can raise the surface pH and can favour their growth. Warmer, more humid regions support Gram-negative bacilli, *S. aureus*, and coryneforms. Sites with high sebaceous gland density (e.g., the face) selectively encourage *lipophilic Cutibacterium* and *Malassezia* [9,38,39,40,41,42,43,44,45,46].

The skin microbiome also depends on host factors such as age, sex, and anatomical location. Delivery mode shapes early colonisation: infants born by Caesarean section acquire predominantly skin-associated taxa, whereas vaginally delivered infants acquire maternal vaginal communities. Puberty markedly alters the microbiome as sebaceous activity increases; prepubescent skin is enriched for Streptococcaceae, Firmicutes, Proteobacteria, and Bacteroidetes with a more diverse mycobiome, while postpubescent skin favours lipophilic *Cutibacterium, Corynebacterium*, and *Malassezia*. With advancing age, community structure continues to evolve and correlates with age-associated dermatoses: staphylococcal atopic dermatitis in childhood, *C. acnes*–associated acne in adolescence, and *Malassezia*-associated tinea versicolor in adulthood. Sex-linked anatomical and physiological differences (e.g., sebum output, barrier features) and environmental exposures (clothing, medications, occupation) add further variance [5,9,44,46,47,48,49,50,51,52,53,54,55,56,57,58,59,60].

High-resolution surveys underscore this topographical and temporal diversity. In a 16S rRNA gene phylotyping study, 51.8% of sequences were assigned to Actinobacteria, 24.4% to Firmicutes, 16.5% to Proteobacteria, and 6.3% to Bacteroidetes. Among 205 genera, *Corynebacterium*, *Cutibacterium* (historically “Propionibacteria”), and *Staphylococcustogether* accounted for over 62% of sequences. Sebaceous sites were dominated by *Cutibacterium* and *Staphylococcus*, moist sites by *Corynebacterium* and *Staphylococcus*, and dry sites by a broader set including β-Proteobacteria and Flavobacteriales [23,61,62].

Cutaneous propionibacteria (now classified within *Cutibacterium*) are commensals of keratinised epithelia and include *C. acnes* (formerly *P. acnes*), *C. granulosum*, *C. avidum*, *C. lymphophilum*, and *C. propionicum*. They are non-motile, Gram-positive, and exhibit coryneform morphology. Historically, these organisms have been grouped with Bacillus, anaerobic diphtheroids, and Corynebacterium. *C. acnes* and *C. granulosum* are typically isolated from sebaceous skin (face, back, upper chest), whereas *C. avidum* is enriched in the axilla [5,18,19,63,64,65].

Although bacteria predominate numerically, the cutaneous mycobiome—notably *Malassezia* spp.—is consistently detected. Smaller studies using 18S rRNA and other markers highlight *Malassezia* restricta, *M. globosa*, and *M. sympodialis* as prevalent isolates, especially in lipid-rich, sebum-dense sites, reflecting their lipophilic metabolism [35,66,67,68,69].

Together, these findings portray the skin as a site-stratified, physiochemically diverse ecosystem. Its resident communities are co-adapted with host structures and defences and vary predictably with life stage, sex, and environment—principles that are essential to understanding dysbiosis and the pathogenesis of acne.

In recent years, the skin has been recognised nor merely as a physical barrier but as a neuro-immune-endocrine interface capable of integrating environmental signals and coordinating systemic homeostasis [70,71,72]. According to recent evidence, the cutaneous epithelium expresses a functional equivalent of the hypothalamic–pituitary–adrenal (HPA) axis, including CRH, ACTH, cortisol, and related receptors, which enable local adaptation to stressors such as UV lights, pollutants, pathogens, and psychosocial triggers [70,72,73,74,75,76,77,78,79]. Keratinocytes, sebocytes, immune cells, and peripheral nerve endings participate in this bidirectional communication network, linking the nervous, endocrine, and immune systems. Environmental stimuli can therefore modulate innate immune activation, antimicrobial peptide expression, melanogenesis, lipid production, and barrier function [76,77,80,81,82]. This neuro-immuno-cutaneous integration is especially relevant for acne, a condition strongly influenced by stress-responsive sebocytes, inflammatory amplification loops, and microbiome dynamics. Incorporating these neuroendocrine pathways is essential for understanding how internal physiology and external exposures jointly shape skin homeostasis [73,76,77,80,81,83].

Objective and Scope

This narrative review provides an updated synthesis of the current evidence linking the skin microbiome to the pathogenesis of acne vulgaris. It also compares synthetic and natural therapeutic approaches, highlighting translational opportunities and emerging biotechnological strategies. By integrating mechanistic insights with clinical relevance, the review aims to outline future directions for microbiome-based acne management.

To achieve this objective, a comprehensive literature search was conducted using PubMed, Scopus, and Web of Science databases, covering the period from 2010 to 2025. The search employed the keywords: “*acne vulgaris*”, “*microbiome*”, “*retinoids*”, “*polyphenols*” and “*Cutibacterium acnes*”. Only peer-reviewed articles published in English that addressed mechanistic or therapeutic aspects were included. Additional references were identified from the bibliographies of key reviews and primary research articles.

Acne

Even though host and microorganisms typically coexist in balance, shifts in host physiology or the cutaneous environment can drive normally beneficial or benign microbes toward pathogenic behaviour. Many common dermatoses are associated with dysbiosis, i.e., alterations in the composition or function of commensal communities. In acne, dysbiosis reflects quantitative and qualitative changes in commensals within the pilosebaceous niche, and accumulating evidence suggests that both individual taxa and the broader microbial community contribute to disease expression [33,84,85,86,87,88].

- Definition and clinical spectrum

**Figure 2:**
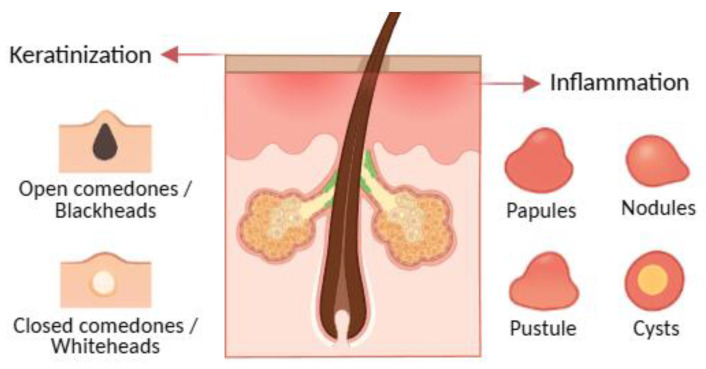
Acne lesions, illustrating comedonal (open and closed comedones) and inflammatory (papules, pustules, nodules, and cysts) types. Created with BioRender.com, with additional AI-assisted graphic design.

Acne is a chronic inflammatory dermatosis of the pilosebaceous unit, historically associated with *Propionibacterium acnes* (now *Cutibacterium acnes*), and characterised by non-inflammatory lesions such as open comedones, more commonly called blackheads, and closed comedones or whiteheads and inflammatory lesions like papules, pustules, nodules, and cysts (Figure 2) [89,90]. The condition involves altered bacterial colonisation within follicles, but pathogenesis is multifactorial. Notably, *C. acnes* is abundant in the microbiota of most adults; however, only a subset develop acne, indicating that strain-level differences, host factors, and micro environmental context modulate pathogenicity. Transcriptional profiles of *C. acnes* also differ between healthy and acne skin, supporting a functional rather than purely abundance-based shift [5,22,33,87,91,92,93,94,95,96].

- Distribution and onset

Lesions predominate at sites with a high density of pilosebaceous units—the face, back, neck, upper chest, and shoulders [97,98]. Acne typically begins in early puberty with increasing sebum output, mid-facial comedones, and subsequent inflammatory lesions; it is commonly seen in girls around 12 years and in boys somewhat later, often from ~15 years. Prepubertal acne can occur and is usually comedonal, reflecting limited sebaceous activity at younger ages [98,99,100,101,102]. Family history and early-onset comedones can help predict severity [99,103,104,105].

Modifiers and triggers Several endogenous and exogenous factors influence onset and flares. Emotional stress, cyclical hormonal fluctuations (e.g., perimenstrual), and excoriation/skin picking can exacerbate disease. Occlusion and comedogenic products, friction and sweat (e.g., sports gear), and certain medications (notably some antiepileptics) can provoke monomorphic eruptions; anabolic-androgenic steroids may induce severe forms. Evidence for sunlight, diet, and hygiene is mixed; counselling should be individualised [106,107,108,109,110,111,112,113,114,115,116,117,118,119,120,121].

- Burden and psychosocial impact

Beyond pruritus, soreness, and pain, acne carries a substantial psychosocial burden. Visible lesions and scarring can cause embarrassment, anxiety, and reduced well-being. Because acne peaks during adolescence—a critical period for identity and self-esteem—minimising scarring and controlling inflammation are central to care; clinicians should avoid trivialising acne as merely self-limited [97,122,123,124,125,126,127].

Pathogenesis: four interlocking processes

Acne arises from the convergence of four tightly connected biological processes within the pilosebaceous unit:

1. Follicular hyperkeratinisation produces microcomedones through abnormal keratinocyte proliferation and desquamation, narrowing the infundibulum and obstructing outflow [128,129,130,131,132,133,134,135].
2. Sebum overproduction and altered composition, driven by androgens and metabolic signals such as IGF-1, creates a lipid-rich, anaerobic niche; sebum output correlates with acne severity [131,134,136,137,138,139,140].
3. Microbial factors—especially *C. acnes* colonisation—promote disease via lipases, hyaluronidases, and proteases that liberate free fatty acids, compromise the barrier, and foster comedogenesis; biofilm formation may shield bacteria from host defences and antibiotics [22,87,131,140,141,142,143,144].
4. Inflammation and innate–adaptive crosstalk amplifies and sustain lesions: IL-1 signalling, neutrophil recruitment and ROS generation, and leukotriene B4–mediated cascades are implicated. Sebocytes synthesize neuropeptides, antimicrobial peptides, and antibacterial lipids, linking stress (CRH axis) and vitamin D signalling to sebaceous activity [22,128,129,130,136,137,140,145,146,147].

**Figure 3:**
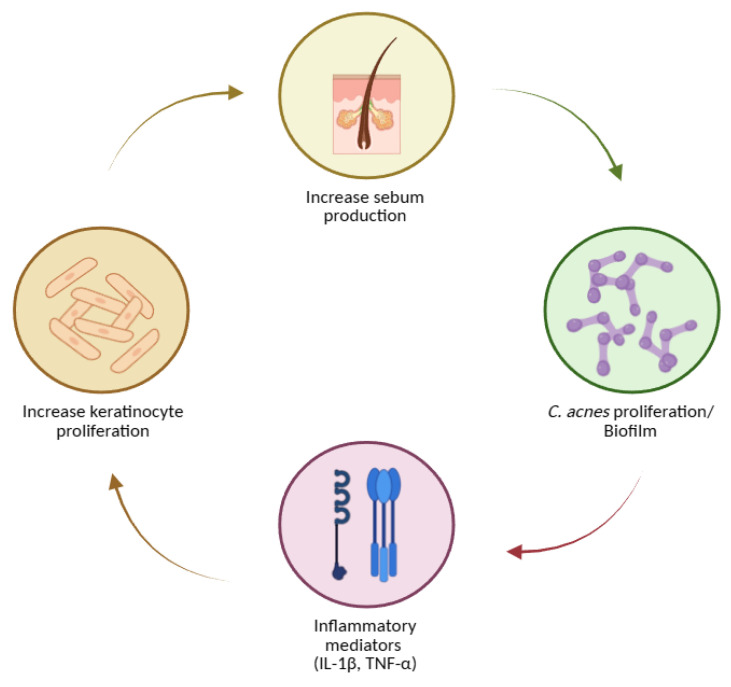
Key steps in acne pathogenesis, including increased sebum production, *Cutibacterium acnes* proliferation and biofilm formation, induction of inflammatory mediators (IL-1β, TNF-α), and enhanced keratinocyte proliferation. Created with BioRender.com.

These processes can be envisioned as a self-reinforcing loop (Figure 3): hyperkeratinisation and altered sebum favour *C. acnes* growth and biofilms; bacterial products intensify inflammation; inflammatory mediators (including IL-1) further dysregulate keratinisation and sebaceous function [128,129,130,136,137,141,142,145,148].

Sebum, comedogenesis, and lesion evolution

**Figure 4:**
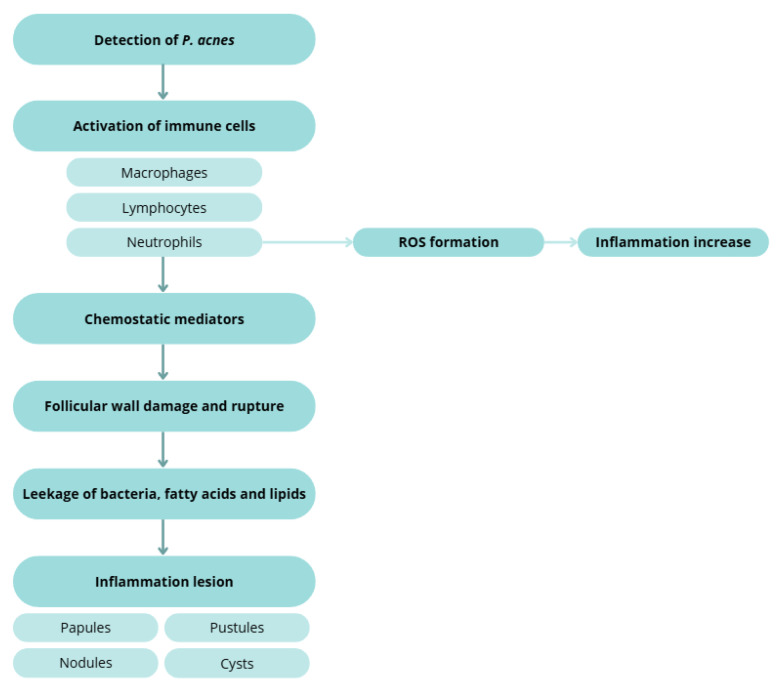
Inflammatory response to *C. acnes*, illustrating immune cell activation, ROS formation, chemo tactic mediator release, follicular wall rupture, and the development of inflammatory lesions (papules, pustules, nodules, and cysts). Created with BioRender.com.

Excess sebum—stimulated by androgens, particularly testosterone—correlates with disease severity [131,139,149]. Hyperproliferation and dysregulated shedding of keratinocytes lead to accumulation of corneocytes, lipids, and filamentous material within the follicle, forming microcomedones that progress to visible open or closed comedones [22,131,132,133,135,150]. In both normal follicles and comedones, microflora typically include *Staphylococcus epidermidis* (coagulase-negative), *C. acnes* and *C. granulosum* (anaerobic diphtheroids), and *Pityrosporum* spp. (lipophilic yeasts). *S. epidermidis*—an aerobic surface commensal—appears less implicated in inflammation than *C. acnes*, consistent with antibody response patterns. In contrast, *C. acnes* thrive in sebaceous follicles and hydrolyses triglycerides to free fatty acids and glycerol, promoting comedogenesis and inflammation (Figure 4) [22,86,87,131,143,151,152].

Classical work on cutaneous endocrinology established the concept of the skin as a stress organ, capable of synthesizing and metabolizing neuro hormones traditionally associated with central endocrine organs. Acute or chronic stress activates cutaneous CHR receptors, inducing sebocyte proliferation, enhancing lipid synthesis, and promoting abnormal desquamation, which are key steps in microcomedone formation. Stress also reduces barrier integrity and shifts antimicrobial peptide expression, facilitating colonization by acne associated *C. acnes* phylotypes [75,78,153,154,155].

Inflammatory cascades and tissue injury

Host detection of *C. acnes* triggers macrophage, lymphocyte, and neutrophil activation. Chemotactic factors and ROS generation contribute to follicular wall damage and rupture with extrusion of keratin, lipids, and bacteria into the dermis, producing papules, pustules, nodules, and cysts [123,131,132,145,156,157].

Inflammation in acne is increasingly understood as a convergence of classical immune pathways with neurogenic mediators. Substance P, CGRP, and other neuropeptides released from peripheral nerve endings stimulate keratinocytes, mast cells, and sebocytes, provoking IL-1, IL-6, TNF-α, and matrix metalloproteinase release. These mediators intensify neutrophilic infiltration and oxidative stress while altering sebocyte lipid profiles in ways that support *C. acnes* proliferation. Neurogenic inflammation therefore acts as an upstream trigger that lowers the immune activation threshold within the pilosebaceous unit [22,135,158,159,160,161].

Melatonin, long known for its circadian and neuroendocrine roles, is now recognized as a key cutaneous antioxidant and immunomodulator. Human skin synthesizes melatonin and converts it to metabolites such as AFMK and AMK, which exhibit strong ROS scavenging capacity, anti-inflammatory activity, mitochondrial protection, and microbiome modulating effects [162,163,164,165,166,167]. These molecules counteract lipid peroxidation within sebocytes, inhibit NF-κB and MAPK signalling, reduce IL-1β and TNF-α expression, and support balanced keratinocyte differentiation. Dysregulation of local melatonin metabolism may therefore exacerbate oxidative stress and inflammatory cascades central to acne pathophysiology. Given the melatonin metabolites originate evolutionary in early organisms such as bacteria and insects, their conserved antioxidant function may reflect an ancient defence system relevant to modern skin disorders [163,164,166,167,168].

Vitamin D signalling is also integral to skin homeostasis and acne development. Active vitamin D (1,25-dihydroxyvitamin D) regulates sebocyte proliferation, suppresses lipid synthesis, and enhances antimicrobial peptide production, particularly cathelicidin (LL-37), which modulates *C. acnes* survival and immune activation [169,170,171,172,173]. Vitamin D additionally downregulates Th1/Th17 pathways and reduces IL-17-mediated inflammation, mechanisms implicated in nodulocystic disease. Dysregulated vitamin D signalling may promote barrier dysfunction, dysbiosis, and heightened inflammation [170,171,172,173,174,175,176].

Strain-level insights

Culture-independent studies show that while overall *C. acnes* abundance may not differ dramatically between acne and controls, specific phylotypes/lineages associate with disease, whereas others are enriched in health. Follicular biopsies from acne patients reveal higher frequencies of disease-linked *C. acnes* strains and a greater proportion of colonised follicles, supporting a strain-selective colonisation model rather than a simple overgrowth model [22,88,93,177,178,179].

These data demonstrate the multifactorially, dysbiosis-linked inflammatory disorder of the pilosebaceous unit in which keratinisation dynamics, sebum quantity/quality, microbial traits (including biofilms and strain diversity), and immune networks intersect to drive lesion initiation, progression, and scarring.

Acne Treatments

Management of acne aims to control active lesions, reduce inflammation, prevent and treat scarring, and minimise relapses. Each treatment must be tailored to the patient’s clinical history and specific needs, considering lesion morphology, severity, distribution, age, sex, psychosocial impact, and prior response [180]. Several therapeutic approaches are available—topical, systemic, and natural/non-drug treatments—and combining agents that target different pathogenic mechanisms generally yields faster and more durable outcomes, as it can be seen in Table 1 [181].

Topical treatments

Topical therapy remains the foundation for mild-to-moderate acne and is available in creams, gels, lotions, and cleansers. While these agents act directly on the affected area, local irritation and dryness may occur [185,188].

Retinoids are first-line agents because they normalise follicular desquamation, prevent microcomedone formation, and exert anti-inflammatory activity [132,182,185,219]. Tretinoin regulates epithelial desquamation, preventing blockage of the pilosebaceous unit and reducing inflammation [122,180,184,185,219,220]. Adapalene, a synthetic lipophilic analogue, penetrates the follicle efficiently, normalises keratinocyte differentiation, and is generally best tolerated for maintenance [122,123,183,185,219,220]. Tazarotene, a pro-drug converted to tazarotenic acid in keratinocytes, modulates proliferation and differentiation and has strong anti-inflammatory properties [122,131,185,219,220]. Retinoids can be used alone in predominantly comedonal acne or combined with antimicrobial agents for inflammatory forms. Gradual titration and attention to vehicle and formulation improve adherence and tolerability. Innovative micro- or nano-delivery systems are being explored to increase potency at the target while minimising irritation [122,123,124,131,132,133,182,183,185,219,220].

**Figure 5:**
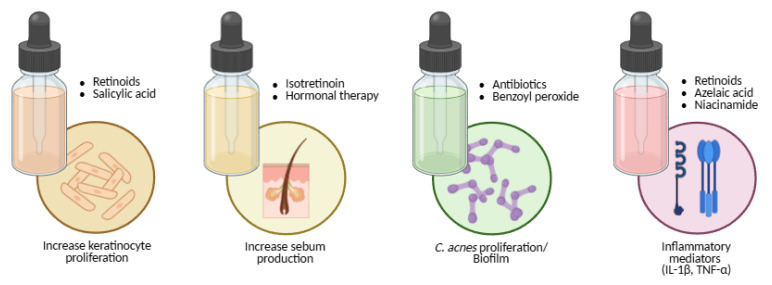
Mapping acne therapies to the core pathogenic pathways: keratinocyte hyperproliferation, sebogenesis, *Cutibacterium acnes* overgrowth and biofilm, and inflammatory mediator release (IL-1β, TNF-α). Created with BioRender.com.

Topical antibiotics reduce *Cutibacterium acnes* and inflammatory lesions (Figure 5). Erythromycin (a macrolide) and clindamycin (a lincosamide) act by binding the bacterial 50S ribosomal subunit, inhibiting protein synthesis [122,180,182,185,205,221]. Resistance to erythromycin has reached approximately 60% in *C. acnes* [180,185,188,221], so both agents should be prescribed only short-term (≈12 weeks) and never as monotherapy. Combining antibiotics with benzoyl peroxide (BPO) or retinoids enhances efficacy and mitigates resistance [185,189,190,191,192,222,223].

Benzoyl peroxide is a potent oxidising and comedolytic agent that kills *C. acnes* independently of resistance mechanisms (Figure 5). It reduces comedones but may cause peeling, dryness, erythema, or fabric bleaching [122,180,193,220,222,223,224]. Azelaic acid offers comedolytic, antibacterial, and anti-tyrosinase effects, making it valuable for post-inflammatory hyperpigmentation and safe in pregnancy (Figure 5) [122,123,180,185,193,198,225].

Niacinamide (vitamin B_3_ amide) decreases sebocyte lipid output and inflammation and strengthens the epidermal barrier (Figure 5) [185,194,195,196,197,199,200,225]. Salicylic acid, a keratolytic agent, dissolves intercellular cement, enhances penetration of co-applied actives, and exerts mild bacteriostatic and fungistatic effects (Figure 5) [122,180,185,225].

Systemic treatments

Systemic therapy is indicated for moderate-to-severe, nodular, truncal, or scarring acne, or when topical management fails.

Oral isotretinoin remains the only drug capable of long-term remission. It induces sebaceous-gland involution, markedly suppresses sebum production, normalises keratinisation, and alters the follicular microenvironment (Figure 5) [123,183,185,201,202,203,204,226,227,228,229,230]. Because of teratogenic risk, strict pregnancy-prevention protocols and laboratory monitoring are mandatory. Counselling should also address mucocutaneous dryness, lipid changes, and mood symptoms [185,226,227,228,229,230].

Oral antibiotics are reserved for moderate-to-severe inflammatory acne or widespread disease [185,192,193,201]. Tetracyclines (e.g., doxycycline) combine antimicrobial and anti-inflammatory activity with relatively low resistance rates [182,185,193,229,230,231]. Macrolides (erythromycin, clindamycin) serve as alternatives when tetracyclines are contraindicated but show higher resistance potential. Fluoroquinolones (e.g., levofloxacin) are discouraged under stewardship principles [132,182,185,192,202,205,229,230,231]. To maximise efficacy and reduce resistance, oral antibiotics should always be combined with topical retinoids or BPO and prescribed for the shortest effective duration [132,182,185,201,229,231].

Hormonal therapy targets androgen-driven sebogenesis. Combined oral contraceptives and anti-androgens such as spironolactone reduce free testosterone and suppress sebaceous activity (Figure 5) [132,182,185,198,201,206,229,230]. They are suitable for adolescent and adult women when hormonal influence is evident, provided contraindications (e.g., thromboembolic risk) are assessed individually [185,229,230].

Natural and adjunct approaches

Natural products treatment

Given the side-effects and resistance issues associated with conventional drugs, natural bioactives have emerged as safe, multi-target alternatives. Therefore, researchers are focusing on natural bioactive options. It has been that these compounds may act differently disrupting distinct metabolic pathways [207]. It is important to mention that natural approaches can influence the hormonal characteristics of the disease, sebum production, inflammation, infection and hyperkeratinization [208].

- Green tea

**Figure 6:**
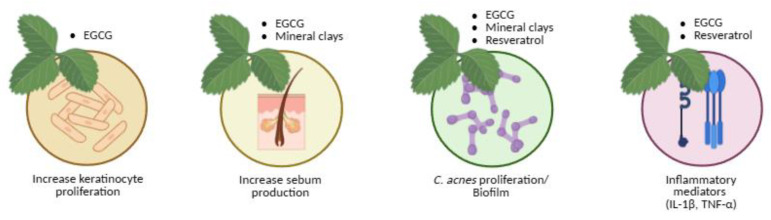
Natural therapeutic options for acne aligned with their corresponding pathogenic targets.

Green tea polyphenols, particularly epigallocatechin-3-gallate (EGCG), display anti-inflammatory, antioxidant, antimicrobial, and sebo-suppressive properties [182,209,232,233]. EGCG targets key acne pathways—reducing *C. acnes* growth, inflammation, and aberrant keratinisation—and clinical studies have shown decreased inflammatory and non-inflammatory lesions after eight weeks of topical use (Figure 6) [210,232,233].

Mechanistically, epigallocatechin-3-gallate (EGCG) mitigates oxidative stress by scavenging reactive oxygen species (ROS) and protecting skin cells from oxidative damage. It also helps regulate sebum production by inhibiting 5α-reductase, thereby reducing androgen-mediated acne. Additionally, EGCG suppresses several pro-inflammatory mediators and signalling pathways, including TNF-α, NF-κB, and PI3K/Akt, while downregulating IL-23 mRNA expression, collectively contributing to its anti-acne efficacy [182,234].

- Mineral clays

Mineral clays (halloysite, talc, sericite, kaolin) have been used since antiquity for acne and blackheads. Applied as masks, they open pilosebaceous orifices, absorb sebum, and promote perspiration [217,235,236]. The therapeutic benefits of clays are attributed to their high adsorption capacity, which allows them to bind sebum, impurities, and toxins while forming a protective film on the skin surface. This action helps shield the skin from external chemical and physical irritants and supports barrier regeneration. By reducing excess oiliness and promoting a cleaner skin environment, clay-based formulations are considered beneficial for acne management [217,235,236,237,238].

In vitro studies have demonstrated that clays can inhibit *C. acnes* and *S. epidermidis*, although clinical outcomes depend on the specific composition and formulation (Figure 6) [218]. For example, a study by Meier [239] evaluated the effectiveness of facial masks containing clay and jojoba oil in individuals with mild acne. Treatment with these masks resulted in reductions in papules, pustules, and comedones, indicating clear clinical improvement.

- Resveratrol

Resveratrol, a natural phytoalexin, possesses anti-inflammatory and anti-proliferative activity and inhibits *C. acnes* [211,212,213,214,215,240,241]. In vitro, it acts bacteriostatically at 50–100 mg/L and bactericidally at 200 mg/L [212]; in pilot clinical trials, topical resveratrol gels used for 60 days reduced pustules, macro- and micro-comedones, and epidermal hyperproliferation (Figure 6) [214]. Research by Dos Santos [242] demonstrated that resveratrol, together with quercetin and gallic acid, can inhibit the growth of *C. acnes* at concentrations of 5 μg/mL. For comparison, the commonly used acne treatment benzoyl peroxide achieved similar inhibition at 1 μg/mL. In animal studies, resveratrol reduced skin swelling by 40% and decreased the levels of IL-1β, a key mediator of acne-related inflammation, by 50%, as well as myeloperoxidase by 35%. These findings suggest that phenolic compounds such as resveratrol may alleviate acne by blocking critical inflammatory pathways, including MAPK and NF-κB, triggered by *C. acnes* [240,241,243,244].

Natural compounds modulate key biological processes involved in acne pathogenesis, including keratinocyte proliferation, sebum production, *C. acnes* proliferation/biofilm formation, and inflammatory mediator release. Created with BioRender.com.

Future acne therapies may benefit from incorporating neuroendocrine targeted compounds such as melatonin metabolites and vitamin D analogues. Their ability to modulate inflammation, oxidative stress, sebocyte activity, and microbial homeostasis positions them as attractive candidates for combination or adjunct therapy [163,245,246,247,248]. Integration of these pathways into personalised treatment algorithms, potentially guided by biomarker profiling or neuroendocrine and vitamin D axis activity, may support more precise, durable, and microbiome friendly acne treatments [245,246,247]. These approaches also align with sustainability and One Health principles by reducing reliance on broad-spectrum antibiotics and supporting endogenous regulatory mechanisms.

Clinical integration and outlook

In practice, effective regimens combine a topical retinoid to normalise desquamation with BPO or azelaic acid to reduce bacterial load and inflammation, adding short antibiotic courses or isotretinoin for severe or refractory disease [185,187]. In women with hormonal influence, anti-androgen therapy provides a targeted adjunct [185,186]. Maintenance (low-irritancy retinoid ± azelaic acid), gentle skincare, and photoprotection reduce relapse [185].

Across all modalities, the guiding objective is to increase therapeutic potency at the target site while preserving tolerability, microbial balance, and long-term safety, in alignment with principles of antimicrobial stewardship [122,123,124,131,132,133,180,181,182,183,186,188,189,190,191,193,201,202,203,205,249,250].

Critical Perspective

Traditional, Synthetic, and Natural Approaches

Current acne management reflects a dual paradigm. On one side, traditional synthetic therapies—retinoids, antibiotics, hormonal agents—remain the cornerstone of evidence-based practice. They are supported by extensive clinical data, predictable pharmacodynamics, and regulatory familiarity. However, their limitations are increasingly evident: antibiotic resistance, irritation, photosensitivity, teratogenicity, systemic toxicity, and microbiome disruption. The overuse of broad-spectrum antibiotics has particularly raised concerns regarding antimicrobial stewardship, as *Cutibacterium acnes* resistance threatens both dermatological and systemic infection control [180,183,185,186,188,189,190,191,192,201,202,203,205,232,249]. Furthermore, patient adherence often declines with chronic irritation, dryness, or psychological burden linked to treatment complexity.

Despite substantial progress in elucidating acne pathophysiology and expanding therapeutic options, clinical translation remains inconsistent. Synthetic agents, such as retinoids and antibiotics, demonstrate the highest efficacy but are limited by irritation, teratogenicity, and the rising threat of antimicrobial resistance [132,182,192]. In contrast, natural bioactives—including EGCG, resveratrol, and clay minerals—show promise for multi-target modulation of inflammation and sebum regulation; however, they lack robust pharmacokinetic data and randomized clinical evidence [204,234,237,238,243,249,251,252].

Bridging these gaps requires integrated approaches that combine the precision of synthetic agents with the biocompatibility of natural compounds, guided by microbiome-based diagnostics and antimicrobial stewardship principles. Future research should prioritize long-term, head-to-head trials, mechanistic clarity, and sustainable formulations that maintain microbial equilibrium.

## 2. Conclusions and Future Perspectives

Acne vulgaris exemplifies the complex interplay between host biology, microbial ecology, and environmental factors. Future acne management should adopt personalized, multimodal, and microbiome-conscious approaches. Artificial intelligence combined with microbiome sequencing can facilitate patient-specific treatment algorithms by identifying microbial signatures predictive of therapeutic response. Additionally, nanocarrier and hydrogel delivery systems can improve the local bioavailability of retinoids and polyphenols while minimizing irritation. These technologies address unmet needs for targeted, sustainable, and patient-friendly therapeutics.

## Figures and Tables

**Table 1:** Comparative overview of acne therapies.

Therapy Class	Representative Agents	Mechanism of Action	Reported Efficacy	Common Adverse Effects	References
Topical Retinoids	Tretinoin, Adapalene, Tazarotene	Normalize follicular keratinization; anti-inflammatory; comedolytic	High efficacy in mild–moderate acne; cornerstone of therapy	Erythema, peeling, dryness, photosensitivity	[122,123,124,131,132,133,180,182,183,184,185,186,187]
Topical Antibiotics	Erythromycin, Clindamycin	Inhibit *C. acnes* protein synthesis via 50S ribosomal binding	Moderate efficacy, enhanced when combined with BPO	Local irritation, bacterial resistance (~60% *C. acnes*)	[180,182,183,185,186,187,188,189,190,191,192]
Topical Agents (Other)	Benzoyl peroxide, Azelaic acid, Niacinamide, Salicylic acid	Antimicrobial, keratolytic, anti-inflammatory, sebum regulation	Moderate efficacy, useful as adjuncts or maintenance	Irritation, dryness, bleaching (BPO)	[122,123,180,185,186,187,193,194,195,196,197,198,199,200]
Systemic Retinoids	Isotretinoin	Reduces sebaceous gland size and sebum output; normalizes keratinization	Highest efficacy for severe/nodulocystic acne; long-term remission	Teratogenicity, mucocutaneous dryness, lipid elevation	[123,183,185,187,201,202,203,204]
Systemic Antibiotics	Doxycycline, Erythromycin, Clindamycin, Levofloxacin	Suppress *C. acnes* and reduce inflammation	Moderate–high efficacy for inflammatory acne	GI upset, photosensitivity, resistance risk	[132,182,185,187,192,193,201,202,203,205]
Hormonal Therapy	Combined oral contraceptives, Spironolactone	Decrease androgenic stimulation of sebaceous glands	Moderate efficacy, especially in adult female acne	Nausea, mood changes, thromboembolism	[132,182,198,201,206]
Natural Polyphenols	Green tea EGCG, Resveratrol	Antioxidant, anti-inflammatory, antibacterial, sebo-suppressive	Clinical studies show 40–60% lesion reduction in 8–12 weeks	Minimal irritation; mild dryness	[182,207,208,209,210,211,212,213,214,215,216]
Mineral Clays	Kaolin, Halloysite, Sericite, Talc	Adsorb sebum, cleanse pores, mild antimicrobial effect	Moderate efficacy; beneficial as adjunct masks	Dryness, transient irritation	[217,218]
Other Natural/Adjunct Approaches	Probiotics, botanical extracts (e.g., niacinamide)	Modulate inflammation and microbial balance	Variable efficacy; promising in small trials	Mild irritation, sensitivity reactions	[194,195,196,197,207,208]

This comparative summary underscores that retinoids remain the most effective acne therapies but are limited by irritation and safety concerns. Antibiotic use is effective yet increasingly constrained by resistance, emphasizing combination and stewardship strategies. Hormonal agents benefit select female patients, while natural bioactives such resveratrol offer promising microbiome-friendly alternatives that require further clinical validation.
